# The mitochondrial genome of *Diaphanosoma dubium* with comparison with *Daphnia magna*

**DOI:** 10.1080/23802359.2017.1413295

**Published:** 2017-12-08

**Authors:** Ping Liu, Shaolin Xu, Qi Huang, Henri J Dumont, Qiuqi Lin, Bo-Ping Han

**Affiliations:** a Institute of Hydrobiology, Jinan University, Guangzhou, China;; b Department of Biology, University of Gent, Gent, Belgium

**Keywords:** *Diaphanosoma dubium*, neodiaphanosoma, mitochondrial genome, recombination

## Abstract

*Diaphanosoma* has been called “tropical *Daphnia*” for its strong ecological role in tropical freshwater as *Daphnia* in temperate waters. The present study sequenced and annotated the mitochondrial genome (MG) of *Diaphanosoma dubium*. The MG of *Diaphanosoma dubium* is 16,362 bp in length, with typical metazoan gene composition. Phylogenetic analysis confirms an earlier finding that *Neodiaphanosoma* can be separated from *Diaphanosoma* as a subgenera. One unknown extra CDS region and different arrangement of tRNA were identified when this MG was compared to that of *Daphnia magna*. This is the first non-daphnia MG of *Cladocera*, and information on MG sequence and tRNA order provide valuable molecular data in understanding phylogeny of *Diaphanosoma* and *Cladocera*.

*Daphnia* is the keystone genus in temperate aquatic ecosystems, and its tropical partner, *Diaphanosoma,* plays a similar role in warm waters and has been called “tropical *Daphnia*” (Dumont 1994; Sarma et al. 2005). Among all *Diaphanosoma*, *Diaphanosoma dubium* is one of the most widely distributed species (Korovchinsky 2000; Liu et al. 2018), and it is the common dominant *Cladocera* in eutrophic lakes and reservoirs in tropics and subtropics (Chen et al. 2011). Its high population density could indicate a strong ecological function. Therefore, *Diaphanosoma dubium* can be a model organism to study effect of global change in tropics and population genetics over large spatial scale. However, few molecular data are available for *Diaphanosoma dubium* and its congeners. Mitochondrial genomes (MG) can increase resolution in phylogeny (Cameron 2014; Schreeg et al. 2016) and provide more genetic information when compared to the frequently used barcoding sequence (COI). Here, we sequenced and annotated the MG of *Diaphanosoma dubium*, and compared it to that of *Daphnia magna*.

Living animal was collected from Liuxihe reservoir (116.20 E, 39.97 N) and massively cultured in small aquarium. Sample is preserved in Institute of Hydrobiology, Jinan University, Guangzhou, China. Thousands of individual was gathered after 1 moth of cultivation, and the collection was preserved in −80 °C before sending to Beijing Genomics Institute (BGI) for next-generation sequencing (Hiseq 2500, PE-150). The assembly and annotation procedure followed Xu et al. (2017) with COI sequence of *Diaphanosoma dubium* (AB549201) as seed. COI of four other *Diaphanosoma* species (*D. excisum*, *D. orghidani*, *D. brachyurum* and *D. mongolianum*) were amplified with 2 pairs of primers: LCO1490 and HCO2198 (Vrijenhoek 1994), CrustF (Costa et al. 2007) and HCO2198. Based on COI sequence of all available Sididae species from NCBI and this study, a maximum-likelihood phylogeny was reconstructed with PhyML (Guindon et al. 2010) in Seaview (Gouy et al. 2010). Finally, the synteny of all mitochondrial genes between *Diaphanosoma dubium* and *Daphnia magna* was analyzed using SimpleSynteny (Veltri et al. 2016).

The total length of *Diaphanosoma dubium* MG (MG428405) is 16,362 bp, with A + T biased composition: A (27.8%), C (16.1%), G (18.2%), T (37.8%). Thirty-seven genes were annotated: 13 protein coding genes, 22 tRNA genes and two rRNA genes. Among all PCGs, ND2 and ND6 are initiated with ATT, COX1 with GTG, and the rest with ATG. Twelve genes end up with complete stop codon: ND2, COX1 and CYTB with TAG, ATP6, ATP8, COX3, ND1, ND3, ND5, ND4, ND4L and ND6 with TAA. Only COX2 end up with a single “T”. tRNA genes have length ranging from 58 to 72 bp, and they are folded into typical cloverleaf structure. The putative control region is 517 bp in length, with high A + T composition (63.4%). The phylogeny for Sididae was in compatible with that from Guo (2015) (Figure 1). *Diaphanosoma* was split into two subgenera, namely *Diaphanosoma* Fischer S.S. and *Neodiaphanosoma* Paggi. When compared with the MG of *Daphnia magna* (NC_026914), *Diaphanosoma dubium*’s MG is 1414 bp longer, which could mostly result from an extra unknown CDS (locate between 1496 and 2473 bp). Moreover, different arrangement of tRNA was found between these two species, and no shift of PCGs was discovered.

**Figure 1.**
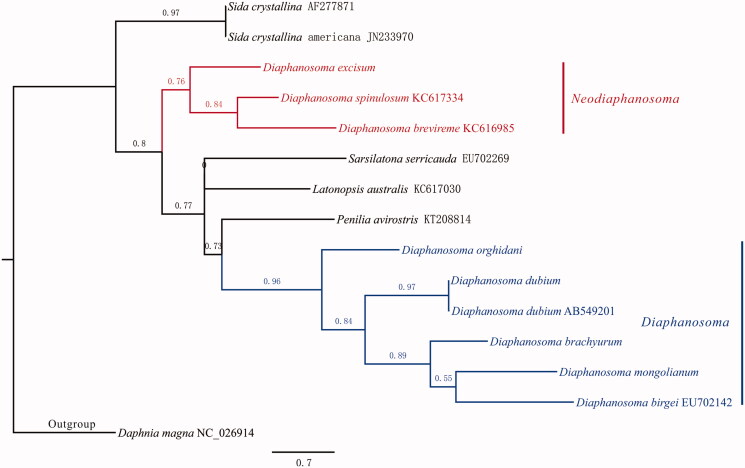
Phylogeny of Sididae. Sequence without NCBI ID are from this study. Parameters in PhyML were set as following: aLRT for Branch Support, optimize nucleotide equilibrium frequencies, invariable site ratio, and selected best of NNI & SPR for tree searching operation, all the rest are left as default.

## References

[CIT0001] Cameron SL. 2014 Insect mitochondrial genomics: implications for evolution and phylogeny. Annu Rev Entomol. 2014. 59:95–117. 24160435 10.1146/annurev-ento-011613-162007

[CIT0002] Chen H, Cheng D, Xu L, Lin Q, Han B. 2011 Distribution of *Diaphanosoma dubium* and *D. orghidani* in reservoirs of Guangdong Province, southern China. J Lake Sci. 23:801–805.

[CIT0003] Costa FO, deWaard JR, Boutillier J, Ratnasingham S, Dooh RT, Hajibabaei M, Hebert PDN. 2007 Biological identifications through DNA barcodes: the case of the Crustacea. Can J Fish Aquat Sci. 64:272–295.

[CIT0004] Dumont HJ. 1994 On the diversity of the Cladocera in the tropics. Hydrobiologia. 272:27–38.

[CIT0005] Gouy M, Guindon S, Gascuel O. 2010 SeaView Version 4: a multiplatform graphical user interface for sequence alignment and phylogenetic tree building. Mol Biol Evol. 27:221–224. 19854763 10.1093/molbev/msp259

[CIT0006] Guindon S, Dufayard J-F, Lefort V, Anisimova M, Hordijk W, Gascuel O. 2010 New algorithms and methods to estimate maximum-likelihood phylogenies: assessing the performance of PhyML 3.0. Syst. Biol. 59:307–321. 20525638 10.1093/sysbio/syq010

[CIT0007] Guo F. 2015 Towards a phylogenetic taxonomy of the genus Diaphanosoma (Crustacea, Ctenopoda, Sididae) based on the sixth trunk limb and on COI gene sequences. Guangzhou: Jinan University.

[CIT0008] Korovchinsky NM. 2000 Redescription of *Diaphanosoma dubium* Manuilova, 1964 (Branchiopoda: Ctenopoda: Sididae), and description of a new, related species. Hydrobiologia. 441:73–92.

[CIT0009] Liu P, Xu L, Xu S, Martínez A, Chen H, Cheng D, Dumont HJ, Han B-P, Fontaneto D. 2018 Species and hybrids in the genus *Diaphanosoma* Fisher, 1850 (Crustacea: Branchiopoda: Cladocera). Mol Phylogenet Evol. 118:369–378. 29107154 10.1016/j.ympev.2017.10.016

[CIT0010] Sarma SSS, Nandini S, Gulati RD. 2005 Life history strategies of cladocerans: comparisons of tropical and temperate taxa. Hydrobiologia. 542:315–333.

[CIT0011] Schreeg ME, Marr HS, Tarigo JL, Cohn LA, Bird DM, Scholl EH, Levy MG, Wiegmann BM, Birkenheuer AJ. 2016 Mitochondrial genome sequences and structures aid in the resolution of piroplasmida phylogeny. Plos One. 11:e0165702 27832128 10.1371/journal.pone.0165702 PMC5104439

[CIT0012] Veltri D, Wight MM, Crouch JA. 2016 SimpleSynteny: a web-based tool for visualization of microsynteny across multiple species. Nucleic Acids Res. 44:W41–W45. 27141960 10.1093/nar/gkw330 PMC4987899

[CIT0013] Vrijenhoek R. 1994 DNA primers for amplification of mitochondrial cytochrome *c* oxidase subunit I from diverse metazoan invertebrates. Mol Marine Biol Biotechnol. 3:294–299. 7881515

[CIT0014] Xu S, Guan Z, Huang Q, Xu L, Vierstraete A, Dumon H, Lin Q 2017 The mitochondrial genome of Atrocalopteryx melli Ris, 1912 (Zygoptera: Calopterygidae) via Ion Torrent PGM NGS sequencing. Mitochondrial DNA B. DOI: 10.1080/23802359.2017.1413307. PMC7800031 33474087

